# A Maximum-Entropy approach for accurate document annotation in the biomedical domain

**DOI:** 10.1186/2041-1480-3-S1-S2

**Published:** 2012-04-24

**Authors:** George Tsatsaronis, Natalia Macari, Sunna Torge, Heiko Dietze, Michael Schroeder

**Affiliations:** 1 Biotechnology Center (BIOTEC), Technische Universität Dresden, 01307 Dresden, Germany; 2 Lawrence Berkeley National Laboratory, Berkeley, CA 94720, USA

## Abstract

The increasing number of scientific literature on the Web and the absence of efficient tools used for classifying and searching the documents are the two most important factors that influence the speed of the search and the quality of the results. Previous studies have shown that the usage of ontologies makes it possible to process document and query information at the semantic level, which greatly improves the search for the relevant information and makes one step further towards the *Semantic Web.* A fundamental step in these approaches is the annotation of documents with ontology concepts, which can also be seen as a classification task. In this paper we address this issue for the biomedical domain and present a new automated and robust method, based on a *Maximum Entropy* approach, for annotating biomedical literature documents with terms from the *Medical Subject Headings* (*MeSH*)*.*

The experimental evaluation shows that the suggested *Maximum Entropy* approach for annotating biomedical documents with *MeSH* terms is highly accurate, robust to the ambiguity of terms, and can provide very good performance even when a very small number of training documents is used. More precisely, we show that the proposed algorithm obtained an average *F-measure* of 92.4% (precision 99.41%, recall 86.77%) for the full range of the explored terms (4,078 *MeSH* terms), and that the algorithm’s performance is resilient to terms’ ambiguity, achieving an average *F-measure* of 92.42% (precision 99.32%, recall 86.87%) in the explored *MeSH* terms which were found to be ambiguous according to the *Unified Medical Language System* (*UMLS*) thesaurus. Finally, we compared the results of the suggested methodology with a *Naive Bayes* and a *Decision Trees* classification approach, and we show that the *Maximum Entropy* based approach performed with higher *F-Measure* in both ambiguous and monosemous *MeSH* terms.

## Background

In this section we provide the background for this study, including our motivation for addressing the problem of automated document annotation in the biomedical literature with an approach that is robust to the ambiguity of the indexed terms. A formulation of the problem is also presented, as well as a summary of the suggested approach and of the reported results.

### Introduction and motivation

With the rapid expansion of the internet as a means of retrieving related scientific and educational literature, the search for relevant information has become a difficult and time consuming process. The current state of the internet data can be characterized by weak structures and, practically, the absence of relationships between them. Current popular search engines, such as *Google* and *Yahoo*, provide a keyword-based search, which takes into account mainly the surface string similarity between query and document terms, and often a simple synonym expansion, omitting other types of information about terms, such as polysemy and homonymy. In order to address this problem and improve search results, the usage of ontologies has been employed in several previous works, that allows for document annotation with domain specific ontology concepts. The usage of ontologies provides a content-based access to the data, which makes it possible to process information at the semantic level and significantly improve the search of relevant documents, as it has been shown by recent studies in the case of the search in the life sciences literature [1,2].

Some representative examples of such search engines for the biomedical domain are: (a) *GoPubMed* (http://www.gopubmed.com/web/gopubmed/) which uses the *Gene Ontology* (*GO*) and the *Medical Subject Headings* (*MeSH*) as background knowledge for indexing the biomedical literature stored in the *PubMed* database (http://www.ncbi.nlm.nih.gov/pubmed/), and various text mining techniques and algorithms (stemming, tokenization, synonym detection) for the identification of relevant ontology entities in *PubMed* abstracts, (b) *semedico* (http://www.semedico.org), which provides access to semantic metadata about abstracts indexed in *PubMed* using the *JULIE Lab text mining engine* (http://www.julielab.de) and *MeSH* as a knowledge base, and (c) *novoseek* (http://www.novoseek.com), which uses external available data and contextual term information to identify key biomedical terms in biomedical literature documents. However, in all aforementioned engines there are several challenges that arise and need to be addressed, in order for the respective systems to be maintained up-to-date; more precisely: (i) the amount of scientific documents to be annotated and indexed is very large, as the number of indexed *PubMed* documents grows really fast, (ii) the presence of ambiguous terms constitutes the classification (annotation) process, and, thus, the indexing process of articles with *MeSH* terms a challenging task, and, (iii) any classifier model being used to annotate the literature documents with *MeSH* terms needs to be trained and tuned specifically for this domain, in order to achieve the best possible results, and in tandem needs to be fast and robust to address challenges (i) and (ii) respectively. In this work we address these challenges and propose a robust method to annotate automatically literature articles with *MeSH* terms, following a *Maximum Entropy*-based approach. The notion behind the *Maximum Entropy*-based approaches is simple and realistic: it is a statistical learning method that models all that is known and assumes nothing about that which is unknown. Thus, given a collection of training instances, it chooses a model consistent with all the instances, but otherwise as uniform as possible. In the section *Approach*, we explain in detail how the suggested *Maximum Entropy*-based approach operates, as well as why we selected it among other learning alternatives, with the most representative reasons being *robustness* and provision of the importance of the features that are most representative for each of the learned classes. This latter property gives interpret ability to the learned models.

#### Growth of the biomedical literature

**Figure 1:**
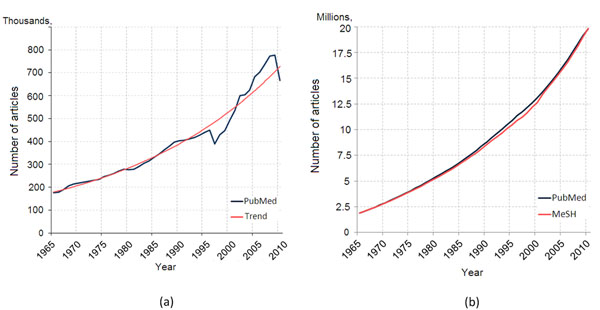
**Trend of indexed PubMed articles and MeSH annotations** In Figure 1(a) we show the number of *PubMed* articles(blue line) indexed over the period 1965 − 2010 and the respective logarithmic trend (red line). In Figure 1(b) the number of *PubMed* articles(blue line) is plotted with the number of the respective *MeSH* annotated documents (red line).

As a proof of concept for (i), i.e., the tremendous growth of the biomedical literature, we present in Figure 1 the growth of *PubMed* indexed documents over the time period 1965 – 2010. The figure shows clearly that new *PubMed* documents are nowadays doubled within the past 20 years (Figure 1(a)), as also discussed in [3]. The exponential trend (red line) also shows that this tendency continues. In parallel, we can observe that the annotated documents with *MeSH* concepts (red line) attempts to keep up with the document growth (Figure 1(b)). For this purpose, the *Medical Text Indexer System* is used (http://ii.nlm.nih.gov/mti.shtml), which makes the annotation process semi-automatic and improves the efficiency of indexing *PubMed* articles compared to a respective manual annotation which would have been impossible given the reported growth. This constitutes as fundamental the need for fast and accurate methods for automated annotation of biomedical literature articles with *MeSH* concepts, so that the growth of *PubMed* documents can be followed with respective *MeSH* terms annotations.

#### Ambiguity analysis of *MeSH* subject headings

As a proof of concept for (ii), i.e., the existence of the ambiguity of *MeSH* terms used to index biomedical literature documents, we performed some measurements with regards to the ambiguity levels of 4,078 *MeSH* terms, which are all the terms under the roots *diseases*, *anatomy*, and *psychology.* In these terms we will also base our analysis and our experimental evaluation. For all of these terms we have measured the number of different meanings that they may carry, consulting three very popular thesauri/lexica, namely the *WordNet* thesaurus for the English language, the *Wikipedia* encyclopedia (English version), which is currently the largest electronic encyclopedia available, and the *Unified Medical Language System* (*UMLS* -http://www.nlm.nih.gov/research/umls/), which is currently the largest available thesaurus for the biomedical domain.

**Figure 2:**
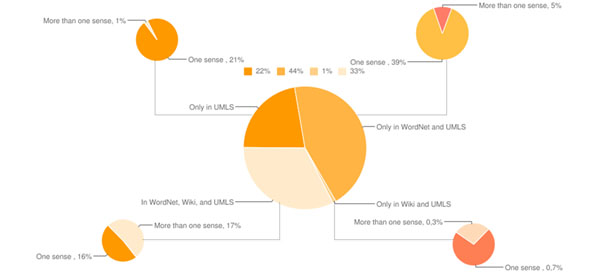
**Ambiguity of MeSH terms** Pie chart showing the ambiguous *MeSH* terms, after examining 4,078 terms, and consulting three dictionaries/thesauri.

The measurements are shown in Figure 2. The figure shows a pie chart with the distribution of the examined *MeSH* terms into polysemous, i.e., ambiguous, and monosemous, after consulting each of the aforementioned thesauri/lexica. It also presents the percentage of terms found in exactly one or in more than one of the used lexica/thesauri. According to the measurements, 23.3% of the examined terms are ambiguous, i.e., they have more than one meaning. Another interesting finding is the coverage of the non-domain specific lexica, i.e., *WordNet* and *Wikipedia*, which is 78% combined. In fact only 22% of the examined have entries only in the domain specific *UMLS* thesaurus.

**Figure 3:**
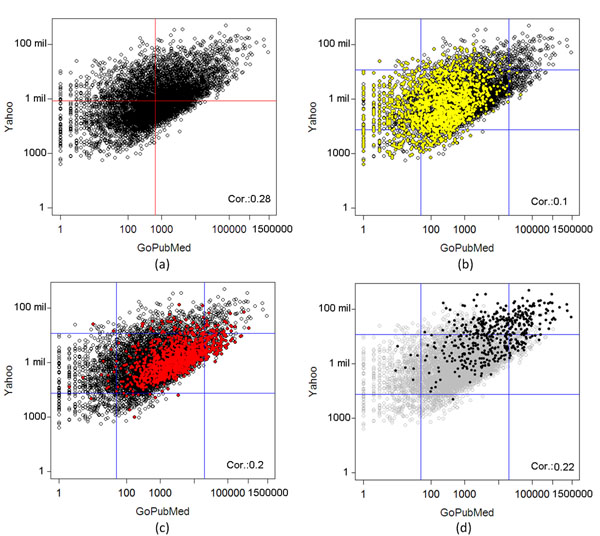
**Relationship between literal appearance and ambiguity of MeSH terms** Scatter plots showing the relationship of the number of documents where the examined *MeSH* terms appear literally in *GoPubMed* (horizontal axis), and in *Yahoo* (vertical axis). Red lines show medians.

In order to stress out the implications of the existence of ambiguous terms in the annotation process, we have furthermore analyzed the number of different documents these 4,078 terms appear literally in *GoPubMed*, as well as in another popular and general purpose search engine, namely *Yahoo.* The aim of this analysis is to show how the number of documents, in which these terms appear literally, varies depending on their number of entries in the used lexica/thesauri. In Figure 3 we present four plots showing the results of this analysis.

Figure 3(a) shows a scatter plot for all of the terms; it plots the number of documents in which each of the examined term appears literally in the *GoPubMed* (horizontal axis) and the *Yahoo* (vertical axis) indexed documents. Comparing the number of results returned by *GoPubMed* and *Yahoo*, the figure shows that their difference in absolute numbers of the retrieved documents is several orders of magnitude. A typical term appears literally in almost 5,000 *GoPubMed* documents, while the respective number returned by *Yahoo* is approximately 1,000,000 documents. The remaining three scatter plots highlight respectively the terms for which there is no entry in the majority of the used lexica (yellow color in Figure 3(b)), the terms for which there is exactly one entry in the majority of the used lexica (red color in Figure 3(c)), and the terms which are ambiguous according to the majority of the used lexica (black color in Figure 3(d)). It is obvious from the plots, that there is a shift of the placement of the terms from left to right and, in parallel, from bottom to top as the number of entries increase, i.e., as we read the plots starting from 3(b) to 3(d). This fact shows that the ambiguous terms may appear in a very large number of documents (contexts), larger compared to the rest of the terms, and, thus, any context-based approach for document annotation will have to handle a lot of noise for these terms, highlighting the need for a very robust annotator.

### Overview of the suggested approach and summary of the results

Taking into consideration the findings of the previous sections, in this work we present a novel approach based on *Maximum Entropy*, that may annotate biomedical literature documents with *MeSH* terms automatically, and with very high *F-Measure.* The approach is supervised and for each of the *MeSH* terms considered it requires a number of positive and negative training examples in order to build a respective context-based model. Thus, the methodology trains a *Maximum Entropy* based classifier for each of the *MeSH* terms, and for the annotation process of an new document, it applies each model to predict whether the respective *MeSH* term should annotate the document or not. The context-based model for each term uses as features terms from the title and/or the abstract of publications indexed by *PubMed*, their publication year, and the name of the journal or forum that that the publication was made. The model for each term retains a list of ranked positive and a list of ranked negative features which was created during the learning process from the positive and negative examples.

Since the problem we are addressing is a multi-label text classification problem, each of the trained models can be applied sequentially, as well as in parallel, as the decision of each of the classifiers does not influence the decision of any of the rest, e.g., a new document may be annotated with any number of *MeSH* terms, without any of the annotations affecting by definition the rest. The results reported from our experimental evaluation show that the suggested method, the details of which are presented in Section *Approach*, may achieve an average *F-Measure* of 92.4% for the full range of the explored terms (4,078 *MeSH* terms). In addition, the results show that the algorithm’s performance is resilient to terms’ ambiguity, achieving an average *F-measure* of 92.42% in the explored *MeSH* terms which were found to be ambiguous according to the *Unified Medical Language System* (*UMLS*) thesaurus.

Finally, we have compared the suggested method with three other approaches, namely: (1) a simple baseline approach which uses exact matching of the *MeSH* terms in the title and/or the abstract of the examined test documents, (2) a *Naive Bayes* classification approach, and, (3) a *decision tree* classification approach. In all cases, the reported results from the compared methods show that our approach produces higher *F-Measure*, both in the set of monosemous, as well as in the set of the polysemous examined *MeSH* terms’ set.

### Related work

Given the formulation of our problem, which if seen from the data mining or machine learning perspective it is a multi-label text classification problem, the options are many. In order to decide for an appropriate supervised learning algorithm, however, to perform the task of classification (annotation of documents with *MeSH* concepts) one has to consider the challenges that are raised and which were analyzed in the previous sections. More precisely an assessment must be made on several criteria such as whether the learner always finds a unique solution, transparency of the produced model, efficiency in cases where the model is built out of complex data (e.g., large number of features), ability of the produced models to handle noise, as well as ability to avoid overfitting, ability of the produced models to handle missing or incomplete data, training and testing performance time, and, finally, whether the method requires the tuning of additional parameters.

In the work of Mitchell [4] analysis of several alternatives can be found that may provide satisfying answers to all or at least many of the above criteria. Some examples of such alternatives may be *Maximum Entropy*, *Hidden Markov Models*, *Neural Networks*, *Bayesian Networks*, *Naive Bayes*, *Decision Trees* and *Support Vector Machines.* Most of the aforementioned approaches may provide in fact a robust unique classification model which is fast in performance (theoretical complexity and execution times). Each of these classifiers, however, may have several advantages and disadvantages in specific applications, and, thus, one has to also consider the fact that in our case we are addressing the specific domain of biomedical documents, and that the task is one of text classification, related with text features.

Considering the above, and given the fact that the *Maximum Entropy* approach has been applied successfully in the past to several natural language and computational linguistic tasks, such as word sense disambiguation (e.g., the method introduced by Doms [1]), part of speech tagging (e.g., the method by Ratnaparkhi [5]), prepositional phrase attachment (e.g., the method by Ratnaparkhi et al. [6]), named entity recognition (e.g., the method by Borthwick [7]), and many more tasks, in this work we decided to adopt the *Maximum Entropy* approach as our supervised learning algorithm for the context models of the *MeSH* terms. We compare, however, the suggested approach in the experimental evaluation with three other methods for the same task, two of which exist in the aforementioned list of popular learners, namely *Naive Bayes* and *Decision Trees* (*C4.5*)*.*

An additional argument for selecting a *Maximum Entropy*-based approach for our task is the fact that similar approaches have been applied to text related tasks in the biomedical domain. More precisely, in [8] Pakhomov et al. apply successfully a *Maximum Entropy*-based approach to extract patient medication status from text, and classify fragments of unrestricted text that exist in biomedical papers to respective patient medication status categories. However the task that they address is different than the one of annotating biomedical documents with *MeSH* terms, from the point of view that it pertains mostly to the ability of a system to extract successfully at a first stage the different mentioned medication status from text, and then attempt to classify the respective fragment accordingly. It, thus, pertains more to information extraction techniques [9], rather than to text classification techniques. In another work, Raychaudhuri et al. [10] apply a *Maximum Entropy-*based approach to annotate biomedical documents with gene functions using terms from *GO.* They report that compared to the *Naive Bayes* approach that they also test, as well as a *Nearest Neighbor* approach, the *Maximum Entropy*-based approach performs better. Our study is complementary to [10] from two perspectives: (i) we evaluate thoroughly the use of other features as well, e.g., title of journal publication and name of the journal, instead of considering solely the terms in the documents’ abstracts, and (ii) we perform our analysis using *MeSH* terms, which were created exclusively for the purpose of indexing biomedical documents for text retrieval, compared to the *GO*, which was created for conceptualizing genes and their functions. Prom this later perspective, the task we address is very different, as these two ontologies, i.e., *MeSH* and *GO* address very different needs, are constructed for totally different purposes, and, thus, their terms carry totally different roles and properties; e.g., as we saw in Figure 2, exactly 23.3% of the examined terms were found to be ambiguous. It is, thus, for the first time to the best of our knowledge that a *Maximum Entropy*-based approach is designed and implemented to annotate automatically biomedical documents with *MeSH* terms.

The most closely related work to this study is probably the work by Trieschnigg et al. [11], who conduct an experimentally study on six different automated *MeSH* classification systems (*MetaMap*, *EAGL*, a language-based model approach, a vector space model approach, a *nearest neighbor* approach and *MTI*)*.*

However, the methodology they follow to create the models for their experimental evaluation is totally different than the one which should be expected for the case of the evaluation of a multi-label text classification system. The idea they use stems from the field of information retrieval. For each candidate *MeSH* term, they build a huge document, let that be *D_i_* for the *MeSH* term *M_i_* that contains all titles and abstracts of papers which have been manually annotated with that term. When a new document arrives that needs to be annotated with the underlying *MeSH* terms, they use a retrieval model to rank each of the *D_i_* documents according to their similarity of the incoming document. Thus, they use the incoming document as a *query* to retrieve the most related of the *D_i_* documents. This process implies that there is a cut-off value in the list of the retrieved documents *D_i_*, which, as they show in their experimental evaluation, is usually sensitive to the number of the training documents used. This methodology is, thus, totally different from the suggested methodology in our work, where we address the problem as one of a multi-label text classification task, and create a trained model for each of the underlying *MeSH* terms by using a selection of negative and positive examples, rather than a problem of text retrieval.

## Results

In this section we provide the details of our *Maximum Entropy*-based approach for document annotation with *MeSH* terms. In addition we present the results of our experimental evaluation giving also an overview of the experimental set up. Several of the methodological details, e.g., with respect to how the positive and the negative examples were chosen and which implementations of the compared algorithms were used, are given in Section *Methods.*

### Approach

The approach that we follow for automated document annotation of biomedical literature documents with *MeSH* concepts creates a context model for each and every concept of the used ontology. Each model is created following a *Maximum Entropy*-based approach, and aims at characterizing the respective terms with using very well indicative features. The *Maximum Entropy* (*MaxEnt*) method is insensitive to noisy data and capable to process incomplete data such as sparse data or data with missing attributes [12]. However, because of sparseness, *MaxEnt* models can suffer from overfitting [13]. Overfitting can be reduced and the performance can be improved with the integration of the *Gaussian prior* into *MaxEnt *[14]. In addition, the *MaxEnt* models can be trained on massive data sets [15], and their implementation is publicly available through open source projects, such as *OpenNLP* (http://opennlp.sourceforge.net/index.html).

*Maximum Entropy* (*MaxEnt*) is a machine learning approach that is used in statistical modelling. The principle of *MaxEnt* is the classification of the testing data into a finite number of classes *A_n_*. We assume that each of the classes *A_i_* is assigned with the probability of occupancy *p*(*A_i_*), where *i* is the index running over all the possible classes. Also, we assume that the sum of the probabilities of all classes equals to 1, i.e., . In the case one of the probabilities equals to 1, it follows that all the others are equal to 0. This is the case where it is known exactly in which class the data is located and, thus, there is no *uncertainty.* The *uncertainty* (also known as *entropy*) is expressed by information that we do not have about the class occupied by the data. Eventually, from a given collection of facts, the probability distribution that leads to the highest value of *uncertainty*, i.e., *maximum entropy*, is selected. For the case of text classification, this can be done for example by the application of the improved iterative scaling algorithm, as described in [14].

In Algorithm 1 we show in detail how we apply *MaxEnt* for the annotation of documents with *MeSH* concepts. The algorithm is separated into two parts: training and testing. For each *MeSH* term we measure the values of pre-selected features by examining *PubMed* documents. The features in our case are of four types: (1) lexical tokens from the titles of *PubMed* documents, (2) lexical tokens from the abstracts of *PubMed* documents, (3) name of the journal in which the respective documents were published, and (4) year of the published documents. The algorithm constructs a context model for each of the terms, trained on a pre-selected set of positive and negative examples. In Section *Methods* we explain in detail how we compute the features, which are the options for selecting the positive and negative examples, and which are the differences in performance between them. For the training part, the weights of the features are maximized using *iteratively reweighted least squares* (*IRLS*)*.* The classes on which the classifier is trained are always two for each constructed model, i.e., for each term: positive, denoted with 1, and negative, denoted with 0. Once the feature weights for each class are maximized and known for each term *m_j_* ∈ *M* (*β_j_*_1_ and *β_j_*_0_ respectively), the testing procedure can be applied, which decides for each term *m_j_* ∈ *M* separately whether it should annotate the instance *t_i_* ∈ *Ts* (positive class), or not (negative class). For this reason, a classification threshold using a parameter *δ* is used. In Section *Methods* we also show how the algorithm behaves for different *δ* values.

### Experimental evaluation and discussion

For our experimental setup we have used 4,078 *MeSH* terms, which are the terms under the *MeSH* roots: *diseases*, *anatomy*, and *psychology* (the list of the *MeSH* terms used, along with all of the reported numbers are provided in additional file 1). The selection of the terms under those roots is not random, as *psychology* is considered to have difficult terms for annotation, because many terms are general, *diseases* is considered to have easy terms for annotating documents, as most of the terms are very specific, and *anatomy* is a category of unknown difficulty. So the selection spans across all levels of expected annotation difficulty.

All of the experiments shown next were conducted using 10-fold cross validation, and in all cases we measure average precision, recall and F-Measure based on the classification results. Table 1 shows the results for our method (*MaxEnt*) as well as of three other methods: (1) a simple baseline technique for annotation, which is the use of exact matching; *Exact Matching* searches for the exact or stemmed appearance of each of the terms in the abstract or the title of the documents and in case it is found, the document is annotated with that term, (2) the *Naive Bayes* classifier, and (3) the *Decision Trees* classifier (*C4.5*)*.*

**Table 1:** Annotation results for the four methods

Method	All Terms			Monosemous Terms			Ambiguous Terms		
	P	R	F	P	R	F	P	R	F
**Exact Matching**	52.3	22.1	23.92	53.73	18.87	21.61	45.48	37.01	34.82

**Naive Bayes**	82.06	97.45	88.79	81.95	97.48	88.72	82.57	97.29	89.09

**Decision Trees**	95.55	79.42	86.06	95.54	79.72	86.24	95.57	78.01	85.2

**MaxEnt**	99.41	86.77	92.4	99.43	86.75	92.39	99.32	86.87	92.42

Results of annotation for the four methods: *Exact Matching*, *Naive Bayes*, *Decision Trees* and *MaxEnt.*

Results on ambiguous and monosemous terms are also shown separately.

In all cases, only the title and the abstract of each document were used for the lexical features. *MaxEnt*, *Naive Bayes* and *Decision Trees* always used the same four feature types described previously. Also, all methods were trained (besides *Exact Matching* which does not need training) and tested always on the same documents. The *δ* parameter for *MaxEnt* was set to the value that was found optimal in the validation set (10% of the training was always kept as validation set), i.e., in our case equal to 0.1. The table shows that our *MaxEnt* approach gives an average F-Measure of 92.4% for all the terms of our experiment, which is almost four times larger than the F-Measure of the Exact Matching approach (23.92% respectively). The other two compared approaches report lower F-Measures from *MaxEnt*, with the *Decision Trees* achieving 86.06% and the *Naive Bayes* just a little higher, at 88.79%. The most interesting observations arise from the separate study of the ambiguous terms, i.e., in this case the terms with more than one entry in *UMLS*, which are included, however, in the results of all terms shown in Table 1. Naturally, the *Exact Matching* approach drops its precision in those terms, compared to its performance in the monosemous terms, by almost 7 percentage points (p.p.), and increases its recall by almost 20 p.p.; *MaxEnt* manages to retain its performance in those terms, compared to its performance in the monosemous. *Naive Bayes* and *Decision Trees* also retain their performance almost at the same levels, compared to the monosemous terms, but their F-Measure remains significantly lower than the F-Measure of *MaxEnt.* Regarding the performance for the individual *MeSH* branches, the *MaxEnt* F-Measure was 93.52% for anatomy, 92.21% for diseases and 91.35% for psychology, which practically verifies our initial assumption about the *psychology* branch, which proved just a bit more difficult to annotate with.

**Figure 4:**
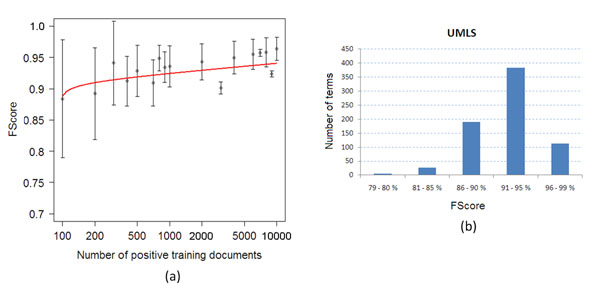
**The effect of the number of training examples and ambiguity in the F-Measure values** The changes in *F-Measure* when number of training examples increases, and the distribution of the performance in *F-Scores* of *MaxEnt* in the ambiguous terms.

In the following we will discuss the performance of *MaxEnt* with regards to how the number of the training examples affects the *F-Measure*, how are the *F-Measure* scores distributed over the ambiguous terms, and how the four features contribute to the overall performance. In Figure 4 we show two plots; Figure 4(a) shows the respective *F-Measure* of *MaxEnt* for increasing number of training documents. As shown, *MaxEnt* can perform really well, even when only few hundreds of training documents per term are used. The figure shows the number of positive training documents, and a respective number of negative training documents is selected (section *Methods* explains how both positive and negative examples are selected). Figure 4(b) shows the distribution of the *F-Measure* values in the ambiguous terms. In the majority of the cases, the *F-Measure* is really high, more than 90%.

**Figure 5:**
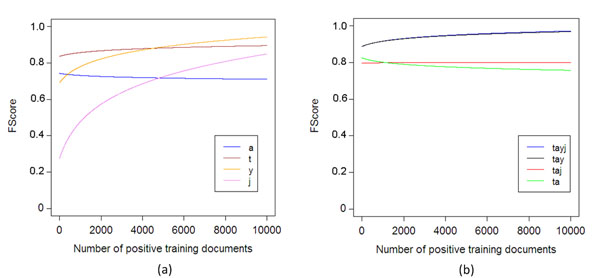
**The contribution of each feature to the annotation performance** Analysis of the contribution of the four features to the overall performance of *MaxEnt.*

In Figure 5 we show an analysis of the contribution of the four different features used to the overall *MaxEnt* performance, again as a function of the number of training documents used. Both figures show F-Measures obtained when using each feature type individually. Figure 5(a) shows the individual contribution of each feature type separately (*abstract*, *title*, *year* and *journal*)*.* As shown, *title* and *year* are the most important features, while *journal* is very important when a large number of training documents is used. The feature *abstract* does not seem to contribute to the overall performance more as the number of training documents is increased, probably due to the fact that the documents’ abstracts contain much context which in the majority of the cases can also contain noise for the purposes of annotating it with the 4,078 terms used. Figure 5(b) presents the *F-Measures* for *MaxEnt* when several combinations of features are explored. The results show again that year is very important (blue and black lines), since, if it is omitted (green and red lines), the performance drops significantly. It is also shown that when all four features used, *MaxEnt* achieves its best *F-Measure*, with little difference in its performance when the *journal* feature is omitted. In the following we give some interpretations and two examples of the reason why the feature *year* turns out to be an important feature for annotating documents with *MeSH* terms. With regards to this feature’s importance, first, it might be the case that a particular *MeSH* term was widely discussed after/before some specific year in time, i.e., the term has a trend in publications during particular years. Therefore, the respective *MaxEnt* model might learn that a certain term is very important during a particular period of time. As a result, the model captures the connections between the term and years of publications. A second explanation would have been that the training examples used for the creation of the *MaxEnt* models had overpopulated a specific time frame. This would mean that the data set used for each term is biased for the respective time period. We ruled out this second explanation as we went over the training examples for each of the 4,078 terms and did not find in any of the cases a significant overpopulation of instances over a specific time frame, i.e., both the used positive and the negative examples cover almost all of the respective *PubMed* documents that were annotated with that term, and, thus, always spanned across large and many different time frames.

**Figure 6:**
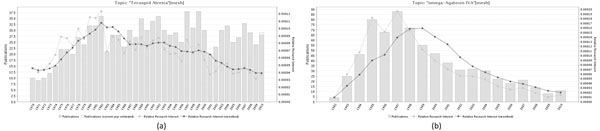
**Examples of MeSH terms’ trends in time** Trend lines of publications over time for the *MeSH* terms *“Tricuspid Atresia”* and *“omega- Agatoxin IVA”.*

Thus, the first explanation is more probable in our case. As a proof of concept for this explanation of the importance of the feature *year* in Figure 6 we plot the trend lines of publications that are annotated with the *MeSH* terms *“Tricuspid Atresia”* (Figure 6(a)) and *“omega- Agatoxin IVA”* (Figure 6(b)) respectively. The smoothed trend line (dark gray line) shows the relative growth of publications in comparison to the growth of the whole *PubMed.* As it is indicated for the term *“Tricuspid Atresia”* most of the publications were conducted before 1999. The term *“omega-Agatoxin IVA”* had attracted much interest before year 2004, but after 2004 and until the end of 2010 respective number of publications decreases. This can definitely explain the fact that the feature *“year after 2004”* can be important indicator of a negative example in this case. Similarly, the feature *“year before 1999”* may appear in the features’ list of the positive examples. Thus, the proposed explanation about the importance of the *year* feature is highly possible due to possible research trends appearing around some of the *MeSH* terms during specific time periods.

Overall, the analysis of the experimental results shows that the suggested *MaxEnt* can annotate documents successfully with *MesH* terms. The results also show that *MaxEnt* produces robust models that are not affected in precision, recall and *F-Measure* by the ambiguity of the terms, and that it performs better than three other annotation methods used, two of which are also popular learners (*Naive Bayes* and *Decision Trees*)*.*

## Conclusions

In this work we introduced a novel approach for annotating documents of the biomedical literature with concepts from the *MeSH* hierarchy. The approach is based on the use of *Maximum Entropy* (*MaxEnt*) classifiers to perform the annotation. For each of the terms, a *MaxEnt* model is trained and it can be applied to any document in order to decide whether it should be annotated with the respective term or not. We performed a thorough experimental evaluation on the application of the proposed *MaxEnt* approach on a selected set of 4,078 *MeSH* terms that were used to annotate biomedical literature documents indexed by *PubMed.* We showed that the used feature types (title, abstract, year, and journal) are sufficient for producing high accuracy annotations. The results showed that the proposed approach was able to annotate automatically *PubMed* documents with an average precision of 99, 41%, average recall of 86.77%, and average F-Measure of 92.4%. As a future work, we plan to investigate role of the *MeSH* hierarchy topology in the annotation results and also experiment on efficient approaches that can combine the annotations of several different types of classifiers.

## Methods

Algorithm 1, described in the *Approach* section, raises several questions regarding the details of its implementation and application for the purposes of this work. In this section we explain these details. More precisely, the following questions are answered: (1) how are the feature vectors constructed, (2) how are the positive and the negative examples selected, and, (3) what is the optimal threshold *δ.*

### Feature vectors

For each *MeSH* term a feature vector may be constructed for each of its associated documents, in order to enable the *MaxEnt* classifier to learn the *β* parameters (feature weights). The associated documents of interest for the *learning step* are of two types: positive and negative, with the former being documents that should be annotated with the examined *MeSH* term, and the later being documents that should not have the examined term as an annotation. For both types of examples a feature vector can be constructed for each document using the procedure described below, the difference between the feature vectors of the training step and the testing step being that the training feature vectors contain an additional feature, which is the class variable (positive or negative), and which is the test cases it is the one that should be predicted.

The feature vectors for each *MeSH* term are of four types: *title*, *abstract*, *journal*, and *year.* Regarding the first two types of the features, they are created with the use of tokenization and stemming procedures. During tokenization, the title and abstract of the article are split into tokens, e.g., words. Next, a stemming procedure is applied, which in our case is *Porter’s* algorithm, which transforms tokens into their morphological root. Next, stop words, i.e., words with low information value like personal pronouns and frequently used verbs are removed from the feature list, and, finally, the remaining tokens are added to the feature list, concatenated with a label, so that we are able to distinguish the features that came from the title, and the features that came from the abstract. The *journal* feature is constructed by the name of the journal, as a string, and a concatenation with a respective label, so that we can distinguish it from the other feature types. For the creation of the *year* feature, which refers to the publication year of the respective document, the words *“****after****”* or *“****before****”* are concatenated with the years starting from 1950 until 2010. Whether *“ ****after****”* or *“****before****”* is concatenated, depends on the publication year of the article. For example, considering an article which was published in 1990, the procedure adds *“****after****”* to the years starting from 1950 till 1990 and *“****before****”* to the years starting from 1991 till 2010. This results to the inclusion of exactly 59 year features inside each feature vector of each term.

### Selection of negative training examples

The algorithm requires an initial set of both positive and negative training examples to construct a *MaxEnt* model for each term. Given a *MeSH* term, the selection of the positive examples is easy, as the manually annotated *PubMed* documents with the specific *MeSH* term can be used (we use all of them, unless they exceed 10, 000, in which case we keep randomly a maximum of 10,000 documents). The challenge is the selection of the negative examples for the training set for each *MeSH* term. For the selection of the negative examples we examined the following three methodologies, which always select documents randomly, equal in number to the positive examples: (a) consider selecting negative examples by randomly selecting documents from all of the available *PubMed* documents, (b) consider only *PubMed* documents that contain literally the *MeSH* term for which the training data set is built, or (c) the subset of (b), for which the abstract content is *semantically distant* from the term. In all cases the documents that are used as positive examples are excluded from the examined set.

For the option (b), the documents were extracted from *PubMed* using the query *” ****term****” ****NOT****”****term****” ****[mh:noexp]***. Such queries retrieve documents that contain the term literally but removes documents with this *MeSH* term. Option (c) is similar to option (b), but requires the measurement of the semantic distance between *MeSH* terms and *PubMed* abstracts. For this purpose we defined our own measure, based on work done in [16], though many more measures exist in the bibliography regarding term similarity or distance in the biomedical domain that can be used [17]. A *MeSH* term is considered to be semantically distant from the *PubMed* abstract if the distance in the *MeSH* tree between the *MeSH* terms of this abstract and the current term is above a specified depth threshold. In our case we set the depth threshold to 6. Thus, we define the distance between two *MeSH* terms *t*_1_ and *t*_2_ as follows:(1)

where *L*(*t_i_*),*L*(*t*_2_) are the minimal depth-levels of the terms *t*_1_,*t*_2_ respectively in the *MeSH* tree, and *CP* is the number of their common parents. The semantic distance between a *MeSH* term and an abstract is then defined as the maximum distance found between the term and any *MeSH* term of the abstract.

Among the three options mentioned ((a), (b), and (c)), experiments conducted showed that there are really small differences in the reported performance, with option (c) producing by a really small margin more robust models. Thus, in the experimental evaluation presented in Section *Results*, all the experiments were conducted with the use of option (c) as a means of selecting the negative examples.

### Classification threshold *δ*

As shown in Algorithm 1, the testing step of the algorithm requires the definition of a parameter *δ*, with which the algorithm can then decide on the positive or the negative class for each new instance, based on the value of the probability produced by the model, and using the value *t* = 0.5 + *δ* as a threshold. In order to discover the optimum *δ*, we have executed experiments with ranging values of *δ* between 0.0 and 0.4. The aim of this experiment was to determine the appropriate accuracy threshold that achieves the best prediction accuracy of the trained models. We altered the parameter *δ* in the validation set from 0.0 to 0.4 and in the vast majority of the cases the results in the validation set were better with *δ* = 0.1. Thus, the reported results in Table 1 use this value for the *δ* parameter.

### Availability of data and implementations of methods

The *MeSH* terms used for our experiments have been made publicly available (please consult the *Additional Files* section on how to obtain them), along with the reported performance of all of the four compared methods. We used a custom implementation of the *Exact Matching* and the *MaxEnt* method, and for the implementation of the *Naive Bayes* and the *Decision Trees* (*C4.5*) methods we used the publicly available out-of-the-box *Mallet* platform (http://mallet.cs.umass.edu/index.php) implementations [18].

## List of abbreviations

*GO:* Gene Ontology; *MaxEnt:* Maximum Entropy; *MeSH:* Medical Subject Headings; *UMLS:* Unified Medical Language System.

## Competing interests

The authors declare that they have no competing interests.

## Authors’ contributions

MS had the original idea. GT, HD and MS designed the algorithm, and NM implemented it, and the *Exact Matching* and executed them in the selected data set. ST implemented the necessary modifications and executed the experiments using *Naive Bayes* and *Decision Trees.* HD also optimized the code of several parts of the experimental process. GT made the ambiguity analysis of the terms, and wrote the main parts of the paper. All authors together analyzed and interpreted the experimental results.

## Authors’ information

Dr. George Tsatsaronis holds a PhD in Text Mining from the Department of Informatics, at Athens University of Economics and Business. He is currently a senior post doc fellow at the Biotechnology center of the Technical University in Dresden, and he conducts research in the fields of text mining with applications in the biomedical domain, word sense disambiguation, natural language processing, and social networks analysis. His research interests also include subjects of Artificial Intelligence, Data Mining and Machine Learning, in which fields, besides his PhD, he also conducted his first post doctoral studies at NTNU in Trondheim, around distributed text mining.

Natalia Macari holds an MSc degree in Computational Logic, from the Informatics Faculty of the Technical University of Dresden. Large part of this work was initiated as part of her MSc project thesis.

Dr. Sunna Torge holds a PhD in the field of Automated Reasoning from the University of Munich. Her main research interests include automated reasoning and controlled natural languages. She has worked for several years within the area of planning and reasoning at Sony International (Europe) GmbH.

Dr. Heiko Dietze holds a PhD on Bioinformatics from the Technical University of Dresden. His work is around semantic enabled technologies for information retrieval in the biomedical domain. His research interests include text mining, natural language processing, algorithms, and software engineering. He is currently working as a post doc fellow in the field of Bioinformatics at the Lawrence Berkeley National Laboratory in USA.

Prof. Dr. Michael Schroeder leads Biotec’s bioinformatics group, in which more than 25 people are working as research scientists. He has published more than 130 scientific papers in top-tier international peer-reviewed conferences and journals within his work fields. He currently holds the position of professor in bioinformatics, at the Faculty of Informatics, Technical University of Dresden.

## Supplementary Material

Additional file 1 **MeSH terms and F-Measures** The reader may wish to consult the file named *“MeSHTermsAndFMeasures.xls”* which contains the *MeSH* ids of the 4,078 terms used for the experimental evaluation, as well as the analytical average *precision*, *recall* and *F-Measure* per term, and four all four methods (*Exact Matching*, *Naive Bayes*, *Decision Trees* and *MaxEnt*)*.* Click here for file
